# Supplementary material for: Complexome profiling on the Chlamydomonas lpa2 mutant reveals insights into PSII biogenesis and new PSII associated proteins
Source: J Exp Bot. 2021 Aug 26;73(1):245–62. doi: 10.1093/jxb/erab390 (PMC8730698; doi:10.1093/jxb/erab390)
Supplement: erab390_suppl_Supplementary_Dataset_S1 [file erab390_suppl_supplementary_dataset_s1.zip › Supplemental Dataset 1 - Excel List and all profiles/plots/PSRP1_Cre05.g237450.html]

Trivial name: PSRP1  
  
Euclidean distance: 10184.58  
Mean Intensity (WT): 656.22  
Mean Intensity (Mut): 432.67  
Distance: 15.52  
  
MapMan: protein.synthesis.ribosomal protein.prokaryotic.chloroplast.30S subunit.S30A;protein.synthesis.ribosomal protein.prokaryotic.chloroplast.30S subunit.S30A  
  
p value of intensity sums Welch test: 0.3590
